# Exploring People’s Reaction and Perceived Issues of the COVID-19 Pandemic at Its Onset

**DOI:** 10.3390/ijerph182010796

**Published:** 2021-10-14

**Authors:** Eamin Z. Heanoy, Ezra H. Nadler, Dominic Lorrain, Norman R. Brown

**Affiliations:** 1 Department of Psychology, University of Alberta, Edmonton, AB T6G 2E9, Canada; lorrain@ualberta.ca (D.L.); nrbrown@ualberta.ca (N.R.B.); 2 Arts and Science Program, McMaster University, Hamilton, ON L8S 4K1, Canada; nadlere@mcmaster.ca

**Keywords:** COVID-19, qualitative, well-being, multi-wave, online comments

## Abstract

The experience of the COVID-19 Pandemic has varied considerably from individual-to-individual. Little is known about the changes in the level of experience general people went through during the first few months after the coronavirus (COVID-19) was declared as a Pandemic. This longitudinal qualitative study explores the general public’s reports of their experience with the COVID-19 Pandemic during its early stage. An online survey was conducted using a convenience/snowball sampling technique in March and again in May 2020, where North American adults with at least a college-degree, and female majority, shared their experiences with the COVID-19 Pandemic in response to an open-ended question, apart from completing questionnaires assessing transitional impact and psychological well-being. Open responses were first content analyzed to identify themes most commonly reported, and then, the quantitative analysis examined the reliability of the changes of themes between the two-time points. Text-analysis of the open-responses from the two waves identified seven themes, namely emotional response, social contact, virus-infected, financial impact, impact on plans, disease, and non-disease related concern, as well as social-distance. These themes indicated that, (a) people were distressed and having negative affective thoughts; (b) they spoke more about their plans-and-goals that were affected by the Pandemic than their financial condition; (c) people mostly used digital platforms to maintain contact with their social network, although they preferred face-to-face interactions; (d) they spoke more about the infection experienced by people in general than infection experienced by themselves and individuals they know. Surprisingly, (e) people mentioned more about the way the Pandemic had disrupted their day-to-day activities than the disease-related health concern. Finally, (f) most of the respondents approved of the practice of social distancing while some expressed its negative or neutral effect on their social lives. The quantitative measure determined that as time passed, people’s experience with the Pandemic became quite different as people talked more about getting infected, and their affected goals-and-plans. We concluded with a remark that this Pandemic would most likely leave an impression on people’s lives and that these online comment-style responses might provide us with insights into people’s perspectives as the Pandemic unfolds, helping us in understanding the uniqueness of the Pandemic experience of individuals for an effective tailored intervention to protect their well-being during a health-crisis.

## 1. Introduction

In December 2019, the world saw the outbreak of novel coronavirus that emerged from China, which was labeled as COVID-19 by the World Health Organization (WHO), and then, it rapidly spread to other parts of the world, making it a global Pandemic [1,2,3,4,5,6]. As of 30 September 2021, there was a total number of 233,503,524 confirmed COVID-19 cases and 4,777,503 total deaths globally, while in North America, the total number of confirmed cases and deaths were 1,629,142 and 27,921 for Canada, and 43,533,168 and 698,672 for the United States (US) [7,8]. The rapid increase of global morbidity and mortality of the COVID-19 Pandemic had raised a significant public health concern along with an economic one. Prior literature on infectious outbreaks demonstrated that this type of public health event commonly invokes distress, fear, and uncertainty, having a profound psychosocial consequence on the individuals [9,10,11,12,13]. The current Pandemic is not different in the sense that it had affected societies and individuals in every facet of life, and the increasing infection/mortality rate, restrictions, prolonged lockdowns, employment situation, and downswing of the economy have had a negative impact on most people’s well-being [14]. 

In March 2020, less than two weeks after WHO officially declared COVID-19 as a Pandemic, several hundred North Americans took part in an online survey, which was designed to assess how the Pandemic was affecting people’s lives during its early stage [15]. This was about the time in North America, specifically in Canada and the US, when classes and office work were moving online, retail establishments were closing, and the Pandemic was coming to dominate the news and social interactions. At that time, it was already clear that the Pandemic was changing peoples’ lives. To document that change, a follow-up survey was conducted in May 2020 where these Canadians and Americans again participated. During both surveys, among other things, they were presented with the following request, “Please provide a description of your ongoing experience of the COVID-19 Pandemic.” Our aim in soliciting these responses was to catalogue people’s unscripted reactions to the Pandemic during its earliest stage and as it unfolded over time. We were particularly interested in learning whether people would express their concerns for their physical, mental, and financial well-being, for the well-being of close others, and for the society as a whole. In addition, we wanted to know whether these reactions would change over time as the novelty of the Pandemic wore off and its enormity sunk in.

This project takes on the Transition Theory [16,17,18,19] perspective as its starting point, which describes that a transition is an event or series of events that causes fundamental changes in the “the fabric of daily life”—what people do, where they do it, and with whom. In addition to the impact on their material circumstances, major life transitions also affect people’s behavior, their mental condition (e.g., their attitude, thoughts, and sense of self), and their physical and emotional well-being [20,21,22,23,24,25,26,27]. Moreover, important transitions typically bring an abrupt end to the regular way of life and lead to a different one [18,19,28,29,30]. From this perspective, the Pandemic has caused a substantial change in people’s personal, social, and economic life as people were already facing financial uncertainty, isolation, concern of being infected, life-activities, and work-related disruption, which created a strong demand for individuals to adjust with this sudden/new transition resulting in a negative well-being outcome [23,31,32,33]. Thus, this Pandemic could be seen as a potentially important, and possibly the largest collective transition, which needed to be documented from its start and followed as it unfolds. Given the unusual feature of it, interpreting only the quantitative findings of the Pandemic studies might not fully reflect individual experience. That being said, in this context, it is necessary to have people express their Pandemic experience in their own words, unconstrained by a quantitative instrument [34].

Pandemic-related studies that mostly included quantitative measures demonstrated a poor well-being outcome of individuals [31,32,35,36,37,38,39,40,41]. Qualitative works were also in line with the quantitative ones, producing similar results, but these studies focused on a specific demographic group such as the frontline workers, caregivers, patients, teachers, etc., and mostly relied on structured or semi-structured interviews [42,43,44,45,46,47,48,49,50]. However, there is a dearth of information on the progressive change in the general public’s behavior, social interaction, thoughts, feelings, or the ways people make sense of what is happening around them during this Pandemic, or at least at the first few months when things were still unknown, collected unsolicited.

In the present study, we intended to explore general people’s experience and issues with the Pandemic and document the dynamicity of their unbidden reaction during the first few months. To the best of our knowledge, this qualitative study is the first to longitudinally capture the general North Americans’ unsolicited response to the Pandemic during its early stage. At the outset, based on prior epidemic-based research [9,10,11,12,13,51,52] and the current COVID-19 research [31,32,33,34,35,36,37,38,39,40,41,42,43,44,45,46,47,48,49,50], we expected that many respondents would indicate in their open-responses that the Pandemic had continued to disrupt their lives by limiting what they could do and where, and interrupting their goals-and-plans. Due to the unprecedented nature of the Pandemic and in the ways that societies have reacted to it (e.g., lockdowns, social distance, crashing financial markets, and high levels of unemployment), we also expected that many would indicate that they found life during the Pandemic to be depressing, anxiety-provoking, and stressful. Furthermore, during the Pandemic, people were going through the unpredictable economic condition, fear of infection, social isolation, and school- and work-related disruptions, and that these issues were related to poor emotional outcomes [31,32,38]. Therefore, we anticipated that people would talk relatively more about these issues as the time passed. For the quantitative analysis, the expectation was that some Pandemic-related issues would be significantly discussed more compared to the other issues during two different time sets, allowing us to have a comprehensive understanding of the Pandemic’s effect [53,54].

We note that there is now a good deal of evidence on the Pandemic and its impact [31,32,36,37,41,55,56,57,58]. However, not all of these articles were available when we conducted the first and second survey and these other studies relied solely on rating-based questionnaire responses. In contrast, the data reported below are derived from open-ended responses and are qualitative in nature. Thus, to the extent that our analyses reveal, people can and do articulate an awareness of the Pandemic’s pernicious effects, providing converging evidence for this important finding, which, in turn, might assist in agencies and professionals’ policy-making decisions on formulating a strategic intervention to provide all-inclusive support, in order to safeguard people’s well-being during a health-crisis. 

On a final note, because of the qualitative nature of the materials collected for this study, they held out the promise of providing unanticipated insights into the ways people were coming to understand the Pandemic during its initial days, which is to say, we treated this project, in part, as an exploratory study and descriptive analysis. Our findings somewhat identified and provided context for understanding the changes in public reaction and concerns about the COVID-19 Pandemic, at least during its initial stage, and suggested that online comment-style open-responses could be a valuable source of qualitative data as such responses might contain a healthy amount of information on not only ‘what’ people think and feel but also ‘how’ [59,60].

## 2. Materials and Methods

### 2.1. Participants

Initially, 1506 respondents from 37 countries completed a web-based survey (i.e., Google Form), which investigated the transitional impact and psychological consequences of the COVID-19 Pandemic [15]. Wave 1 took place from 24 March 2020 to 30 March 2020, less than 2 weeks after the WHO declared COVID-19 to be a global Pandemic. Most Wave 1 respondent were Canadians (942) or Americans (273), and thus, we decided to restrict our analyses to data collected in North America. These people were the general adult population, the majority of which was female, whose day-to-day language was English, and most of them having at least a college degree.

Wave 2 was initiated 8 weeks later with only Canadians and Americans, in order to keep consistency with the first wave. Data were collected between 18 May 2020 to 25 May 2020. At that time, the lockdown was easing in most parts of Canada and in the US. The second wave of data collection took place only with the Canadian and American sample who participated in the first wave. Participants (600 out of 1215) who explicitly stated during the first wave that they would be interested to take part in the follow-up were sent the Google Form to the email addresses they provided. Three-hundred seventy-five participants out of 600 returned the questionnaire, and the remaining 225 did not fill out the survey form; we anticipated the reason was that the participation was strictly voluntary and there was no remuneration for taking part in the follow-up study. The final sample of the second wave consisted of 299 Canadians (79.7%) and 76 Americans (20.2%). Demographic characteristics of the first wave and second wave samples are described in Table 1. Our main purpose was to explore how the experience of these Canadians and Americans with the Pandemic changed over the first few months after the WHO declaration; thus, we limited the capacity of this article to report the qualitative outcome (i.e., open responses) of this study. 

### 2.2. Materials

During both waves, respondents at first completed the 10-items COVID-Transitional Impact Scale (COVID-TIS, a modified version of the TIS-12) [27], the 21-items Depression, Anxiety and Stress Scale (DASS-21) [61], and a 5-point scale Infection-concern rating (details including reliability and validity of these scales have been reported elsewhere [15]). They were then offered the opportunity to answer an open-ended question, which asked the respondents the following question: “Please provide a description of your ongoing experience of the COVID-19 Pandemic”. No word limit was imposed on these open-responses, and they ranged from single-word answers to a full paragraph. Our current report is restricted to these open-ended responses collected in both waves. The final data set consisted of answers to the open-ended question provided by the 375 respondents who completed both Wave 1 and Wave 2. For Wave 2, the total number of open-ended responses was 374 as one of the respondents did not provide any answers in response to the open-ended question.

### 2.3. Procedure

A convenience/snowball sampling strategy was used to recruit respondents. This strategy was implemented by advertising for the first survey through academic channels (e.g., institution email lists, websites), and on social media. The advertisement contained an URL link to the Google form survey that participants were able to complete at a time of their choosing. At the end of this first survey, participants indicated their willingness to take part in a follow-up and, thus, these people were contacted during the second survey and requested to complete the Google form at their convenience. Only people who were 18 years or older were eligible to participate and participation was strictly voluntary; respondents were not compensated in any way for their cooperation. Ethics approval was obtained from the Research Ethics Board of the University of Alberta (Pro00099336).

### 2.4. Text Analysis

An inductive approach was taken to code the Wave 1 and Wave 2 responses because the COVID-19 Pandemic presented us with a novel situation [50]. The data were analyzed following the content analysis approach [62,63]. The goal was to have the themes/codes emerge from the data. Specifically, two co-authors initiated the coding process by reading the open responses from both waves multiple times and making initial observations on groups of words or sentences that in some way provided an identical or relevant meaning of a concept. While making these observations, they tried to be reflexive and avoid their own opinion from affecting the data. These groups of words or sentences were then condensed and coded. The coding process occurred independently as both co-authors separately went through all responses and coded based on the best fit. These separate lists of codes were then discussed and merged into a single final list of 24 codes in total, based on the consensus between the two coders. These codes were classified/named into sub-themes according to their resemblance. Finally, these sub-themes were compared to each other and sorted into main themes. At any point when deemed necessary, clarification of a concept and adjustment to the codes/themes was conducted collaboratively by the co-authors.

To assess coder subjectivity or inter-rater reliability, 10% of the responses from each wave were coded by two other separate coders who were not involved in the initial coding. The agreement between the coders for Wave 1 responses was 80.2% and for Wave 2, it was 80.6% with an aggregated mean percentage of 80.4%. Any discrepancies between the coders were resolved by discussion and coming to an agreement.

This approach yielded 7 key/main themes that captured the main topics, issues, and feelings people wrote about when they shared their Pandemic experiences. The sub-themes (24 in total) referred to repeatedly mentioned topics or opinions; they also reflected the directionality of a comment (i.e., whether it was a positive or negative statement). Table A1 (Appendix A) lists the themes and subthemes that emerged from this process.

## 3. Results

Percentages of mentioned data for the 7 key themes and 24 sub-themes are presented in Table A1 (Appendix A). These data provided a sense of themes that were most important to our respondents during each wave. Below, we described each theme and discussed how these themes and their sub-themes varied over time. Quotes that best represented each theme are presented in Table 2.

### 3.1. Emotional Responses to the Pandemic

Unsurprisingly, respondents often mentioned their emotional reaction to the Pandemic and typically indicated that these reactions were negative. When they elaborated, their comments made clear that they were disturbed by the fact that regular life had come to an abrupt halt and that this required them to make rapid adjustments to the changing circumstances. In addition, our respondents appeared to be bothered by a lack of reliable information concerning COVID-19, and by the increased uncertainty about the future. Overall, this situation led people to mention that they were depressed, anxious, and stressed; in some cases, people also felt hopelessness, fear, frustration, and concern, as they seemed to think that, with each passing day, they were losing control of their lives. In both waves, people typically expressed negative emotion about the Pandemic, although this tendency subsided slightly with time. Some had mixed feelings about the Pandemic, though ambivalent responses were less common during Wave 2. Interestingly, a few people had positive feelings about the Pandemic. These individuals viewed the Pandemic as a means of re-evaluating their morals and beliefs, resilience, humanitarian approach, etc. Some were neutral about this Pandemic, and this neutrality tended to increase during the second wave because, with time, people might have become accustomed to the “New Normal” brought on by the Pandemic.

### 3.2. Social Contact

Many of the responses we collected concerned the Pandemic’s social impact. In particular, many respondents were troubled by the fact that restrictions brought on by the Pandemic reduced their ability to “get together” with friends and family members. This concern, however, was less pronounced during Wave 2 than Wave 1. During Wave 1, those who indicated that they were in regular contact with people outside their homes often mentioned that they relied on the telephone and social media to stay in touch. Again, people mentioned this fact less often in their Wave 2 responses. This shift might indicate that people became more concerned with their own situations and less concerned with contacting others. Alternatively, after several months of social distancing, lockdown, and communicating over digital platforms, people might have got habituated to this “new normal” and, thus, felt no need to mention it. Unsurprisingly, when people did compare electronically mediated communication to face-to-face interaction, they preferred the latter.

### 3.3. Infected with the Virus

This theme subsumes two others: responses by people who indicated that they have been infected with the COVID-19 virus, and responses by individuals who claimed to be acquainted with people who have been infected or who have learned about COVID-19 patients through the media. Predictably, over time, COVID-19 infections were mentioned more often in the Wave 2 responses than the Wave 1 responses. It turned out that in both waves, participants were more likely to discuss infection rates for people in general than infections experienced by identifiable individuals. We assumed this reflected the extensive media coverage the Pandemic received during its early phase [18,19,20]. In addition, in Wave 2, people appeared to have worried more about the health of family members and friends than about their own health. Elsewhere, we have speculated that this asymmetry occurs at least in part because people assume that they could control their own risk-related behavior, but not the risk-related behavior of others [15]. Finally, we note that in both waves, especially compared to Wave 1, Wave 2 responses included more comments about the infections among more distant acquaintances than among family and close others. This reflected the fact that social networks extend far beyond one’s circle of family and friends [21,22], and hence, more people in one’s social network who could become infected.

### 3.4. Financial Impact

Respondents sometimes expressed concern for their own financial condition and for the financial condition of others. Furthermore, some individuals mentioned economic instability caused by the Pandemic in more general terms. Unsurprisingly, respondents who had been laid off or who had lost their jobs often worried about their living situations. We could speculate that this issue would have been more prominent than it was if the US and Canadian governments had not provided aid with programs like Coronavirus Aid, Relief, and Economic Security (CARES), Coronavirus Relief Fund (CRF), Canada Emergency Response Benefit (CERB), etc.

### 3.5. Impact on Plans

This theme concerns the effect, mostly the negative effect, the Pandemic had on people’s goals and plans. Some respondents focused on the Pandemic’s immediate impact, mentioning that their previously planned activities (e.g., academic studies, work-related tasks, travel, vacations) had been disrupted or canceled. Others took a more long-term perspective and considered how the Pandemic might affect their long-term goals (e.g., their careers or domestic situations). As with the themes mentioned above, there was a tendency for the plans-and-goals responses collected during Wave 2 to seem more urgent than those collected during Wave 1; a simple explanation might be that the Pandemic engendered a fair amount of unpredictability about what would happen next, and when life would go back to its pre-Pandemic state. Finally, we note that a few individuals mentioned that their plans had not been disrupted by the Pandemic and that they seemed somewhat surprised by this fact.

### 3.6. Additional Concerns

In addition to the issues discussed above, the open-ended responses in both waves sometimes indicated that both important and mundane activities were curtailed or impeded by the COVID-related restrictions. Among these were: shopping for groceries, going to the gym, dining out, attending cultural events, walking around the park, visiting friends or other people’s places, showing up for medical appointments, etc. Importantly, this non-illness sub-theme about day-to-day activities was more pronounced in the Wave 2 comments than in the Wave 1 comments, suggesting that people were becoming increasingly aware of the negative impact the Pandemic was having on their daily lives. Apart from that, people said that they were more worried about their close others and associates getting infected than themselves contracting or passing the virus to others, but these comments were less prominent than the comments about mundane life activities in both waves.

### 3.7. Social Distancing

Across both waves of data collection, when respondents addressed social distancing, they indicated that they approved of the practice because they believed it was likely to decrease the spread of the virus. However, some people also pointed out that there was a downside to social distancing. Here, the concern was that this policy would have a negative effect on people’s social lives by placing limits on in-person communication and social outing. Of course, some of the respondents who mentioned social distancing were neutral to the practice, though neutral comments were less common during Wave 2 than during Wave 1.

### 3.8. Quantitative Measure

As this is apparent in Table A1 (Appendix A) and as mentioned above, some themes were more common in the Wave 1 responses than in the Wave 2 responses, while others were more common in Wave 2. We wanted to compare the subsequent changes in the proportion of the themes between two time periods. Here, we used Marginal Homogeneity test to determine the reliability of these changes, and to conduct this test, we quantified the sub-themes of each key theme into nominal numbers (e.g., 1, 2, 3, 4) first. For example, the face-to-face sub-theme in the key theme of social contact was coded as 1. In brief, the following themes appeared reliably more often in the Wave 1 responses: maintenance of social contact (MH statistic = -4.04, *p* < 0.001), perceived financial impact (MH statistic = 3.32, *p* = 0.001), illness and non-illness related concern (MH statistic = −7.68, *p* < 0.001), and emotional valence for social distance (MH statistic = −3.46, *p* = 0.001). At the very beginning, introduction to several measures (e.g., lockdown, social-distance, remote-working) to control the Pandemic disrupted people’s typical daily lives. Thus, during the adjustment to this novel situation, they were unable to do routine activities (e.g., grocery, gym), social outings, met their peers and co-workers, and were concerned about getting infected in addition to financial uncertainty, causing people to talk more frequently about these issues. In contrast, these themes were mentioned more often during Wave 2: references to self or others being infected (MH statistic = 4.75, *p* < 0.001), and impact on immediate future plans (MH statistic = −3.00, *p* = 0.003). One speculation could be that, after some time, people might have somewhat adjusted to the Pandemic life. Moreover, they were still living in the same pre-Pandemic place with the same family members, spending time within the same social circle (even virtually), all of which might have factored in declining the frequency of COVID-related issues people mentioned during the first wave. On the other hand, with the increased infection rates and precariousness of their goals-and-plans, people talked more about these issues while conversing after two months compared to the very early stage of the Pandemic. Interestingly, the theme feelings about the Pandemic appeared somewhat at a similar rate across two waves (MH statistic = 0.11, *p* = 0.91), meaning even after few months, people were still as much as apprehensive and conscious as they were at the beginning. In sum, it seems that as time passed, people’s experience with the Pandemic became quite different from their initial response at the very beginning. People talked more about being infected with the virus and their affected future than their social contact, economic condition, concern about the disease and other issues, and their feelings about social distance over time. On the contrary, people’s general feelings about the Pandemic were as similar as they were at the outset.

## 4. Discussion

The qualitative analysis of the responses regarding participants’ experience of the COVID-19 Pandemic revealed that even a couple of months after the first set of data, people’s reaction to the Pandemic was quite intense. Throughout their responses, people talked about different issues such as their feelings about the Pandemic, concerns related to the disease and life activities, feelings about social distancing, maintaining social contact, impact on their future plans, infected with the virus, and financial impact. These seven themes, extracted from people’s responses, provided us with an understanding that even after two months after the first wave of data collection, people still seemed to be uneasy about the COVID situation. One explanation for this could be that their lives continued to be disrupted by this Pandemic. The open responses revealed that, mostly, people were distressed as the Pandemic brought a great deal of uncertainty about the future [38,40,64], and throughout the Pandemic, people had strong feelings of fear, helplessness, etc., due to the unknown nature and psychological demand it caused to adjust with the novel situation [48,65]. Furthermore, with the measures of lockdown, isolation, restriction, etc., the Pandemic had continued to moderately affect participants’ education, career, employment, and their immediate goals-and-plans by creating unpredictability of what would happen next, leaving people to face psychosocial challenges. Interestingly, people were generally positive towards social-distancing practice, but over time, they started to view this as an obstacle to their social relations as they also mentioned that they preferred face-to-face interaction during social communication. People also talked more about close others and associates getting infected than themselves due to persistent fear of contracting the virus because of its highly contagious nature, consequences, etc. [46,48]. All these findings are parallel to other studies about the COVID-19 experience [42,43,44,45,46,47,48,49,50]. However, those studies were conducted in a single wave, using an interview format with special populations, such as front-line workers, COVID patients, etc. In contrast, our study took a multi-wave, open-response approach with the general population, which served to increase its external validity. Additionally, the current method is important as it offered an in-depth understanding of the progressive change of people’s thoughts and attitudes towards the Pandemic [60,66]. Furthermore, in contrast to the traditional interview, the online comment-type format used here granted people the opportunity to choose their own topics to discuss [51]; thus, this comment-style response set-up allowed us to understand people’s decisions on selecting and talking about issues that matter most to them, the trend we also observed in our qualitative outcome. In addition, the quantitative comparison allowed us to have a somewhat comprehensive insight into how people had been experiencing the Pandemic by statistically determining the significant change in the themes or issues between two-time points.

The themes emerging from the open-ended responses highlighted several interesting points. First, the responses revealed that people were distressed and experiencing strong feelings of fear, sadness, and helplessness at the outset of the Pandemic, and these feelings did not dissipate during the two months that separated Wave 1 from Wave 2 [37,67]. At the time when this article was written, it was unknown when the Pandemic era would end or what post-Pandemic life would look like. This concern for an unforeseen future might, in part, had made people apprehensive. Second, people spoke less about their financial condition than their immediate or long-term plans. This was unanticipated given that the job market was unstable, and many workplaces were closed or were forced to lay off staff [31,32]. Of course, it was also true that Americans and Canadians were cushioned against the financial hit by federal policies that provided monetary aid. In contrast, people tended to discuss their short-term and long-term goals and plans in a great deal, perhaps because the Pandemic had an immediate visible impact due to the restriction and changes it brought. As a consequence, it appears to have produced a fair amount of uncertainty concerning the duration of the Pandemic and the state the world would be in when it was over. Finally, it is interesting that people spoke more often about the way the Pandemic had affected the mundane activities (e.g., shopping, grocery, physician visit, exercise, outing, etc.) than about their COVID-related health concerns; this is surprising given the severity of the disease. One explanation could be that repeatedly facing obstacles while engaging with everyday activities might have made them salient issues, whereas the disease itself was not an immediate issue except for people who were infected or who were direct caregivers. As for the quantitative analysis, it informed us that people’s experience and reaction is dynamic in nature as at the very early stage of the Pandemic, social contact, financial impact, diseases, non-disease related concern, and social distance were salient topics of conversation, while after some time, the topics of discussion were more about infection and impact on plans.

Given the distinctiveness of this COVID-19 Pandemic, understanding particular experiences of people demonstrating distress could provide a guiding framework for intervention. The findings of this study might assist in formulating a tailored distress-specific intervention to improve individuals’ overall well-being during this type of public health crisis; this might not only have the potential of dealing with the current Pandemic but also the long-term value of addressing any future one.

### Limitation

Despite the benefits that come from using open responses, the approach we took here does have its downside. One limitation was that responses were collected within a short period of time, using a single question rather than a pre-test suite of questions. Moreover, ideally, face-to-face or telephone interviews should be conducted in order to obtain a robust, precise set of data [49]. Given the time constraints, we were simply unable to develop and pilot a more traditional semi-structured interview. In the current sample, more than 20% of the participants lost their job compared to the 15% in Wave 1, which might have an influence on the responses (more negative in nature). Additionally, we adopted a convenience/snowball sampling strategy due to the time-sensitive nature of the COVID-19 outbreak, which resulted in an oversampling of a certain network of peers (e.g., students and academics), leading to selection bias. That being said, as our analysis was restricted to North Americans, specifically Canada and the US population (i.e., female, college-educated), the scope of generalization of the study outcome is limited beyond this sample. In addition, the time interval between the two waves was relatively short, e.g., two months, while the Pandemic-experience after a year would be much different compared to the initial stage; this long-term experience of the participants might be an important route to explore in the future. Nonetheless, the present study did provide many insights into people’s outlook on the Pandemic, the issues they experienced, and their reaction as the COVID-19 Pandemic progressed.

## 5. Conclusions

To the best of our knowledge, this qualitative report is the first to longitudinally document the shift in general people’s reaction and experience of the COVID-19 Pandemic at its onset. Most qualitative data are collected retrospectively, but the current article provides people’s perspectives and attitudes about the Pandemic from its early days when measures such as lockdown, restriction, distancing, etc., were initiated and it was, overall, an unfamiliar situation. The responses offered some insight into changes in the topics that people were discussing and variability in their perspective over the course of the Pandemic. Findings presented the pattern of feelings and concerns people had over time, and the issues they regarded most important to discuss, pointing out that this Pandemic would leave a distinct mark on people’s lives regardless of its level of impact. The post-Pandemic scene was somewhat unclear at the time of this writing, but we believed that there would be a long-term effect of the COVID Pandemic as sometimes people’s initial and belated response to a crisis might differ when the crisis continues to affect their lives; this is because some adapt with a fairly small adjustment while others struggle with major life changes. Thus, it is important to gain a deep understanding of people’s perception as the Pandemic unfolds, and online comment-style open responses might be a useful resource, apart from the traditional qualitative interview, given the benefit of assessing people’s viewpoint in real-time. Finally, conducting a qualitative analysis followed by a quantitative comparison provided a more nuanced picture of how people experienced the first couple of months of the COVID-19 Pandemic than using only a qualitative or quantitative approach.

## Figures and Tables

**Table 1:** Demographics characteristics of North Americans in first (w1) and second (w2) wave.

Demographic Variable	Statistics (w1 = 1215)	Statistics (w2 = 375)
**Age (*M*, *SD*)**	40.17 (15.83)	40.20 (15.70)
**Gender (*n*, %)**		
Female	930 (76.5%)	292 (77.9%)
Male	272 (22.4%)	79 (21.0%)
Other	13 (1.1%)	4 (1.1%)
**Education level (*n*, %)**		
Less than high school	9 (0.7%)	3 (0.8%)
Highschool or equivalent	212 (17.4%)	51 (13.6%)
Associate	113 (9.3%)	26 (6.9%)
Undergraduate	394 (32.4%)	127 (33.9%)
Graduate or above	487 (40.1%)	168 (44.8%)
**Job (*n*, %)**		
Job loss	187 (15.4%)	83 (22.1%)
No job loss	1028 (84.6%)	292 (77.9%)

**Table 2:** Example of responses representing each theme.

Theme	Responses
**Emotion about the Pandemic**	“Patience, thinking about the community in a larger sense. Trying to stay safe and help those around me. Keeping a positive mind set. Compassion for myself and others.” “COVID 19 has disrupted my entire life. I am frustrated, overwhelmed, and I just want things to return to normal.” “Biggest challenge is dealing with ethical dilemmas, like whether taking an Uber across town is putting myself and others at risk. Evaluating and adhering to the honor system has been a bit exhausting. On the upside, this has created a greater sense of community and we are all in this together-ness, which had kept me from feeling sorry for myself.” “Social Distancing and Change of Lifestyle are only differences I’ve experienced this far. I do not know of anyone in my circle that has contracted the disease.”
**Maintaining social contact**	“I’ve experienced very little change in my social activity. I still tolerate occasional visits from my very small circle of friends. We are careful.” “Similar introverted activities, but no time spent with friends has impacted me, in that I feel a desire to see friends and family more often. I have spent more time on social media interacting with friends and family (i.e., Group FaceTime, etc.).” “I find I really miss my physical activity such as squash. It’s a very social activity also. I’m glad my adult kids are nearby, and I can visit with them. I’m making more phone calls to extended family outside of my home city.” “Major changes to my job and sense of community that make me feel very alone as I live by myself and cannot socialize at work or with my network of friends.”
**Referring self or others being infected**	“So far, I know no one that has been infected but watching the numbers rise higher and higher has me concerned that will change soon. “The majority of my experience has been through media exposure. My family is not close to me and, last night, my mom told me that three of her colleagues at her health clinic have tested positive for COVID-19.” “My son had covid19 (no complications) and I’ve had one friend die from it. I have had mild symptoms of COVID-19 for 9 days, so I have been self-isolating alone.”
**Perceived financial impact**	“I’m an entertainer, I lost all of my jobs and have no work in the foreseeable future. It’s stressful and I’m concerned, but we all need to do our part to keep the curve as flat as possible.” “I have moments of feeling panicked about the health of my family but am mostly incredibly stressed about the financial ramifications of this pandemic. My spouse owns a small business and I work in education. I’m uncertain due to government cuts prior to the pandemic whether I will have a job, and that extra uncertainty is causing me great anxiety.”
**Impact on plans & goals**	“My whole life was finally getting somewhere, I had feelers out for a possible job that might be mine once someone retired, and now I’m worried it’s going to disappear due to attrition and budget restrictions. We were going to buy a house this spring, now I’m thinking we should hang on to our down payment in case one or both of us is unemployed for months. I want kids so bad, but how can I justify bringing a child into the world if I don’t know if I’m going to be able to feed myself in a year? This pandemic has ruined my plans, and that really sucks.” “It hasn’t changed my life much. I have an office job and all of my work can be performed almost seamlessly over a virtual network. In fact, I’ve enjoyed working from home. It has saved me 2 h of commuting and has freed up my mornings and evenings to spend more time on personal development.”
**Additional concern**	“So careful while doing daily activities (e.g., going to supermarket, interacting with roommates) and always checking whether I have physical symptoms such as coughing, etc.” “I know of friends who have been directly impacted and I am very concerned about bringing it home to my mother who is 80. I don’t go out unless I absolutely have to.” “I am mainly concerned about my family members who are at risk—my grandparents and my father-in-law, who is immunocompromised and also undergoing chemotherapy. I am also more wary of those around me when I am at a park or the grocery store, for example, as I do not know where they have been.” “Things have changed so rapidly. I miss being able to go for walks to get coffee and go for dinner or to the movies, or just to people watch. I’m trying to be more active however, and even though I’m tired at home, I’m doing yoga and exercising more than I ever did before.”
**Emotional valence for social distance**	“Actively social distancing/isolating for the good of society and my own health.” “I am extremely extroverted and very touchy-feely and am struggling to do things as I am no longer able to ‘recharge’ through physical affection (hugs, hi-fives, etc.) from friends and family. I also get bored easily despite access to the internet/books, etc., and have no motivation to do any of my schoolwork or dissertation research.” “I was self-isolated due to a coworker’s interaction with someone who tested positive for COVID-19. Luckily, my coworker tested negative, so I am now just social distancing.”

## Data Availability

The anonymous raw data supporting the conclusions of this article will be made available after the authors have completed a multi-wave data collection protocol and have published their findings.

## Appendix A

**Table A1:** Percentage of each theme and its sub-theme for first wave (w1 = 375 responses) and second wave (w2 = 374 responses).

Theme	% of ALL Comments in *w1 **	% of ALL Comments in *w2 **	Sub-Theme	% of Comments within a Theme in *w1 ***	% of Comments within a Theme in *w2 ***
**Emotion about the Pandemic**	100%	100%	Positive	8.78%	8.82%
			Negative	55.85%	52.67%
			Both	18.88%	15.78%
			Neutral	16.49%	22.73%
**Maintaining social contact**	35.11%	33.42%	Face-to-face	22.73%	20.16%
			Electronic	24.24%	12.90%
			Both	3.03%	4.84%
			Limited or no contact	50.00%	62.10%
**Referring self or others being infected**	16.49%	24.87%	Common people	58.06%	60.22%
			Acquaintance	16.13%	17.20%
			Friends & Family	9.68%	13.98%
			Self	16.13%	8.60%
**Perceived financial impact**	11.44%	5.88%	Financial condition affected	58.14%	50.00%
			Concern for self/others financial condition	41.86%	50.00%
**Impact on plans and goals**	22.87%	25.67%	Some change or possibility of change	54.65%	58.33%
			No impact	45.35%	41.67%
**Additional concern**	59.84%	53.21%	Self getting infected	16.44%	18.59%
			Passing the virus	10.67%	6.53%
			Close others and/or associates getting infected	35.56%	21.61%
			Non-illness (e.g., grocery shopping)	37.33%	53.27%
**Emotional valence for social distance**	37.87%	35.29%	Positive	61.97%	60.61%
			Negative	19.01%	23.48%
			Neutral	19.01%	15.91%

* The denominator of the percentage is the total number of responses in each wave; ** The denominator of the percentage is the total frequency of the sub-themes under each theme.
